# Scientific Accuracy

**Published:** 1886-05

**Authors:** 


### SCIENTIFIC ACCURACY.

It is very important that in the discussion of scientific problems we should be exact in statements. In his admirable article on "Aqua Calcis," in this number, Dr. Friedrichs quotes Prof. W. H. Morgan as follows: "Take rice, which has $\frac{91}{100}$ of one per cent. of saline matter in it, and if you sit down and make a simple calculation, there is more bone-making material in rice, if a man were to consume a quarter of a pound a day, than is necessary to build up and make the best osseous structure that a man ever had."

We had previously read this assertion, and given it credence without making the "simple calculation" referred to, but in reading the proof of "Aqua Calcis," that the statement might be verified or disproved, we put pencil to paper and find as follows: The proportion of the quantity of rice named would furnish only about nine-tenths of a pound per year. The bones of an average man weigh about twenty-four pounds, so that it would take more than twenty-six years for the system to accumulate sufficient material for the bony system, to say nothing of the daily waste and repair and the lime salts present in other portions of the body, if the bones were made up exclusively of the earthy salts. This is not the case, but we will allow the figures to stand.

We agree with Drs. Morgan and Friedrichs in their deductions, but we wish them to be drawn from correct premises. The truth is, that one-fourth of a pound of rice would not be sufficient for the subsistance of an average human being, and even if it were possible to make this the only food, it would form but a small part of the source whence the organization could derive the materials from which to elaborate lime salts. A large amount of organic matter is taken in with the fluids that form our drink, and there are even other sources.

